# Glossary of healthcare pathways: a methodological approach involving a transdisciplinary team in public health

**DOI:** 10.3389/fpubh.2024.1347774

**Published:** 2024-04-05

**Authors:** Laurie Fraticelli, Elise Verot, Hans-Martin Späth, Marine C. Genton, Cédric Kempf, Celine Clement, Adeline Darlington-Bernard, Sylvain Roy, Claude Dussart, Gérard Mick, Florence Carrouel

**Affiliations:** ^1^Health, Systemic, Process, UR 4129 Research Unit, University Claude Bernard Lyon 1, University of Lyon, Lyon, France; ^2^Presage Institute, University Jean Monnet, Saint-Etienne, France; ^3^CIC 1408 Inserm, CHU of Saint-Etienne, Saint-Etienne, France; ^4^Laboratory Interpsy, UR4432, University of Lorraine, Nancy, France; ^5^Hospices Civils of Lyon, Lyon, France; ^6^CHU Grenoble-Alpes-Voiron, Voiron, France

**Keywords:** public health, care pathway, glossary, Delphi consensus method, dictionarie, transdisciplinary communication, transdisciplinary research

## Abstract

**Introduction:**

The healthcare pathway is at the heart of public health organization concerns, but communication between the various players can be an obstacle. This work, produced by a French transdisciplinary team, offers a methodological approach based on formalized consensus to elaborate a glossary of healthcare pathways. A two-steps procedure was elaborated, including a double rounded Delphi method to formalize expert consensus, and two groups of experts: a workgroup and a review group.

**Methods:**

The workgroup provided a list of words or expressions that, in their opinion, described, evaluated or compared the healthcare pathways for patients, caregivers or regulators. The review group checked this list and added or deleted words or expressions. Then, definitions were added by the workgroup based into account three dimensions: official, academic and from the field. The review group validated the definitions and provided complementary proposals if needed.

**Results:**

After pooling the list of words proposed by each of the six members of the working group, 417 words/expressions were ranked. After the two rounds of evaluation, 294 words/expressions were rated “appropriate” and were analyzed by the review group. This group, after two rounds of evaluation, agreed on 263 words/expressions that were transmitted to the working group who defined them. These definitions were rated by the review group. The first round of evaluation established 195 definitions as being appropriated whereas 68 definitions were amended by the review group.

**Conclusion:**

This glossary supports transdisciplinary communication, reduces the extent of variations in practice and optimizes decision-making. International debate on all aspects might be strengthened by an improved understanding of the concept of health pathway.

## 1 Introduction

The definition of care pathway was proposed by Vanhaecht et al. (1) as “a complex intervention for the mutual decision-making and organization of care processes for a well-defined group of patients during a well-defined period” (1). In 2005, the European Pathway Association defined the care pathway as a methodology for the mutual decision-making and organization of care for a well-defined group of patients during a well-defined period (2). To characterize a care pathway, it is necessary: (i) to include an explicit statement of the goals and key elements of care based on evidence, best practice, and patients’ expectations and their characteristics; (ii) to facilitate the communication among the team members, with patients and families; (iii) to coordinate the care process by coordinating the roles and sequencing the activities of the transdisciplinary care team, patients and their relatives; (iv) to document, monitor, and evaluate variances and outcomes; and (v) to identify the appropriate resources.

A care pathway aims to enhance the quality of care across the continuum by improving risk-adjusted patient outcomes, promoting patient safety, increasing patient satisfaction, and optimizing the use of resources. This definition involves interdisciplinarity and optimal collaboration between healthcare professionals, patients and researchers. Nancarrow et al. identified 10 characteristics underpinning effective interdisciplinary team work (3) such as communication strategy and structures, appropriate resources and procedures, appropriate skill mix, clarity of vision, quality and outcomes of care, and respecting and understanding roles.

In practice, healthcare teams vary dramatically in their structures and effectiveness in ways that can damage team processes and patient outcomes (4). But how can researchers from various disciplines work together on the understanding of health pathways if they do not use the same semantics? As an example of prior work, a glossary of culture in epidemiology was produced (5) to address three primary classes of challenges; definitional, theoretical and methodological, hypothesizing that culture was a determinant of population differences in health and well-being.

In France, as in many other countries, the pathway approach is at the heart of public health organization concerns. This organization has emerged as a result of the growing increase in chronic diseases and the ageing of the population in developed countries. It aims to standardize the evolution of treatments, reduce the pressure on the healthcare system and the problems related to the areas that require attention by decision-makers (6). In theory, the pathway approach has a number of benefits: (i) reducing the length of the care production process, (ii) enhancing the cohesion of care, (iii) lowering the risk of errors, (iv) reducing the cost of the care production process, (v) enhancing the patient and professional satisfaction (7, 8). Nevertheless, in practice, the introduction of the care pathway concept in the field of public health still falls short of its potential, particularly in terms of promoting transdisciplinarity between healthcare professionals (9). Thus, an obstacle could be the lack of common semantic between the various players. This article, produced by a French transdisciplinary team, offers a glossary to enrich and contribute to this collective dynamic.

The aim of this study was to present a replicable methodology to define key concepts and terms relevant to health pathway, taking into account three dimensions: theoretical, institutional and applied. To our knowledge, this work represents the first initiative driven by a transdisciplinary team composed of healthcare professionals and academic researchers with a replicable and formalized methodology of expert consensus, based on two expert groups. The result of this study was edited in a French glossary intended for a wide audience of healthcare professionals, students and researchers in the field of the healthcare pathways.

## 2 Materials and methods

### 2.1 Study design

A two-steps procedure was specifically elaborated for the purpose of this study, including a double rounded Delphi method (10, 11) to formalize expert consensus, and two groups of experts: a workgroup and a review group.

**Figure 1:**
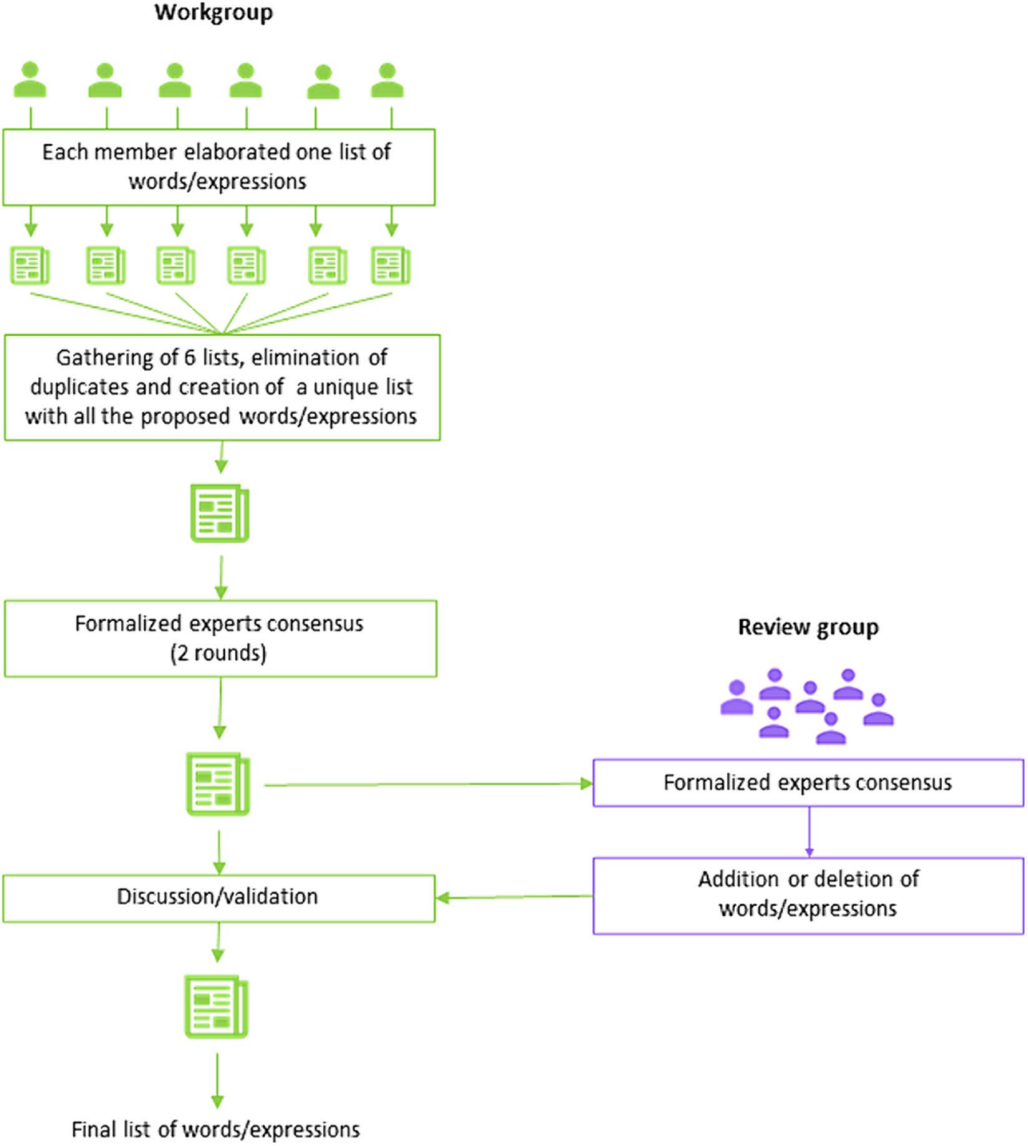
First step of the procedure specifically elaborated for the selection of the words/expressions of the glossary of health pathways.

The first step consisted in choosing the words or expressions to be included in the glossary (Figure 1); the work group had to provide a list of words or expressions that, in their opinion, described, evaluated or compared the healthcare pathways for the benefit of patients, caregivers or regulators. The review group checked the list and added or deleted words or expressions.

**Figure 2:**
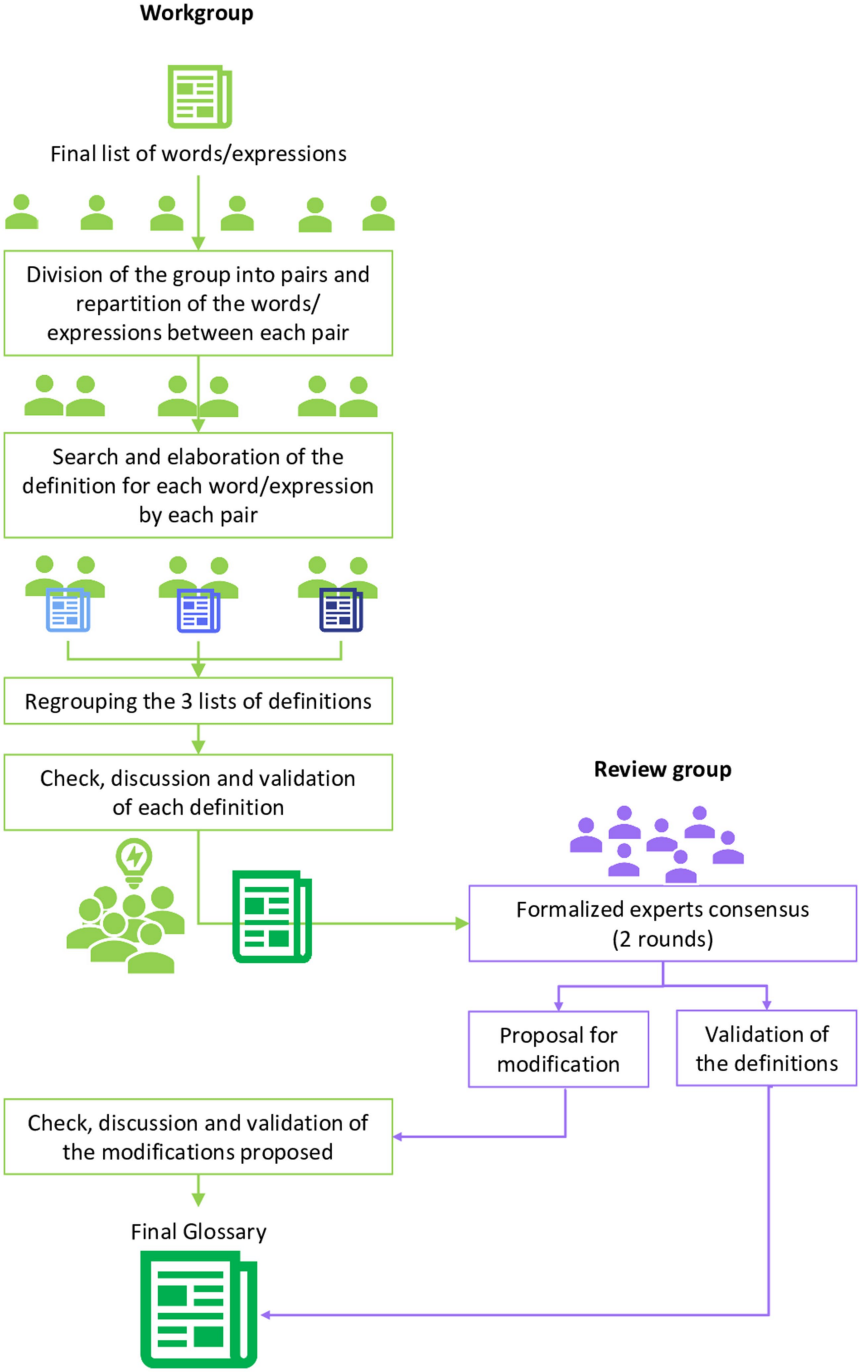
Second step of the procedure specifically elaborated for the definitions of the word/expressions of the glossary of health pathways.

The second step consisted in providing the definitions from the approved list of words and expressions (Figure 2); the workgroup had to search for the most appropriate definitions for each word or expression. The review group validated the definitions and provided complementary proposals if required.

### 2.2 Groups of experts

#### 2.2.1 Workgroup

The workgroup was composed of six volunteers, associated or academic researchers of the laboratory “Health Systemic Process” (P2S, *Parcours Santé Systémique* of the University Claude Bernard, University Lyon 1); a practitioner specialized in neurology, a psychologist, a nurse specialized in tertiary prevention, a pharmacist specialized in health-economy, a specialist of oral health and an epidemiologist-methodologist. The workgroup aimed to constitute a list of words or expressions and to provide definitions.

#### 2.2.2 Review group

The review group was composed of seven volunteers, associated or academic researchers of the laboratory “Health Systemic Process” (P2S, *Parcours Santé Systémique* of the University of Lyon 1); a pharmacist, a specialist of public health, a specialist of pathway for children in difficulties, a specialist of educational science, two specialists of health promotion and prevention and a specialist in oncology pathways. The review group aimed to check and amend the list and the definitions of words or expressions elaborated by the workgroup.

### 2.3 Doubled rounded Delphi and rating analysis

**Figure 3:**
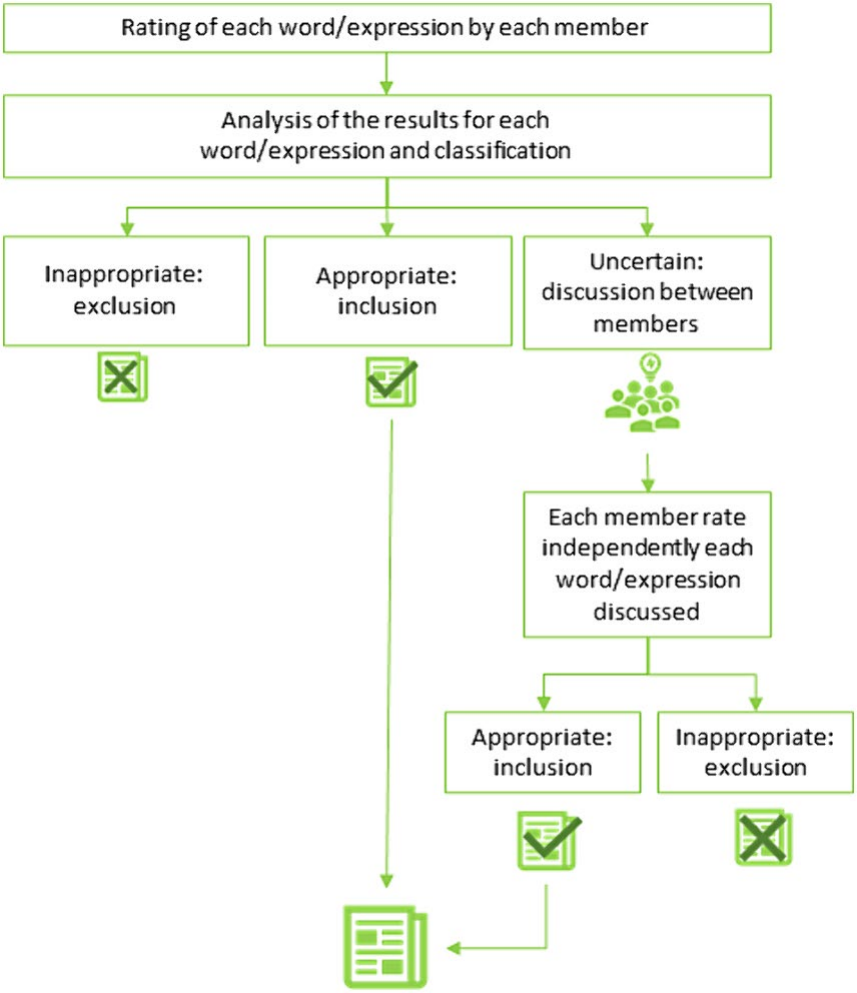
Formalized expert consensus.

The consensus methods are defined as a way to synthesize information and compare contradictory opinions, with the aim to define the degree of agreement within a group of selected individuals. The design of this study was based on the Delphi consensus method to obtain a final, unique, convergent opinion of the group, as described by the recommendations of the Department for good professional practice of the *Haute Autorité de Santé* (HAS, French high authority for health) (Figure 3) (10).

Each member of the workgroup established a list of words or expressions which were potentially related to the characterization or the evaluation of the health/care/life pathway. Then, the members pooled all their proposals and organized a first round of rating to determine if the words/expressions should be included in the glossary. Each word/expression was rated from 1 (totally disagree) to 9 (totally agree) with 5 to express indecision. The ratings were analyzed according to the rules described in Table 1. If a word/expression was deemed appropriate, then it was included in the glossary. If a word/expression was deemed inappropriate, then it was excluded. If the word/expression was considered to be uncertain or when a value was missing, the members of the workgroup discussed and rated it for a second round. If an agreement was still not reached, then the word/expression was rejected.

**Table 1:** Conditions for obtaining an agreement between experts according to median value and distribution of the quotations.

Proposal estimated	Degree of agreement	Median	Minimum; Maximum
Appropriate	Strong	≥ 7	7; 9
Appropriate	Relative	≥ 7	5; 9
Inappropriate	Strong	≤ 3	1; 3
Inappropriate	Relative	≤ 3.5	1; 5
Uncertain	Indecision	4 ≤ median ≤ 6.5	1; 9
Uncertain	No consensus	All other situations	All other situations

### 2.4 Structure of the definitions

The glossary was composed of all the words and expressions retained by the workgroup and the review group: each of them was defined in several sections organized as follows:

– Etymology: this section stated the origin of words and the way in which their meanings changed throughout history. For expressions, the etymology of each individual word was associated. French dictionaries constituted the main etymological source.
– Current dictionary definition: this section provided the definition from the most recent version of the French language dictionary (*Larousse*) for each word defined independently.
– Definition from an official source: when applicable. Sources in this section were extracted from official sources, such as public, governmental or institutional websites, from legal texts, such as health programs. When the definition retained was a legal text, the definition was verified by a lawyer.
– Academic definition: sources included works from the national academy of medicine, the University Press of France or the National Library of France. Also, French scientific articles published in peer-reviewed journals were used.
– Definition from the field: this section reported definitions from patient or health professional associations, hospital centers or regional agencies for health.
– Associated terms: this section referred to other words/expressions related to the words/expressions defined in the glossary.

## 3 Results

### 3.1 Result of the process leading to the list of words/expressions to be defined

**Figure 4:**
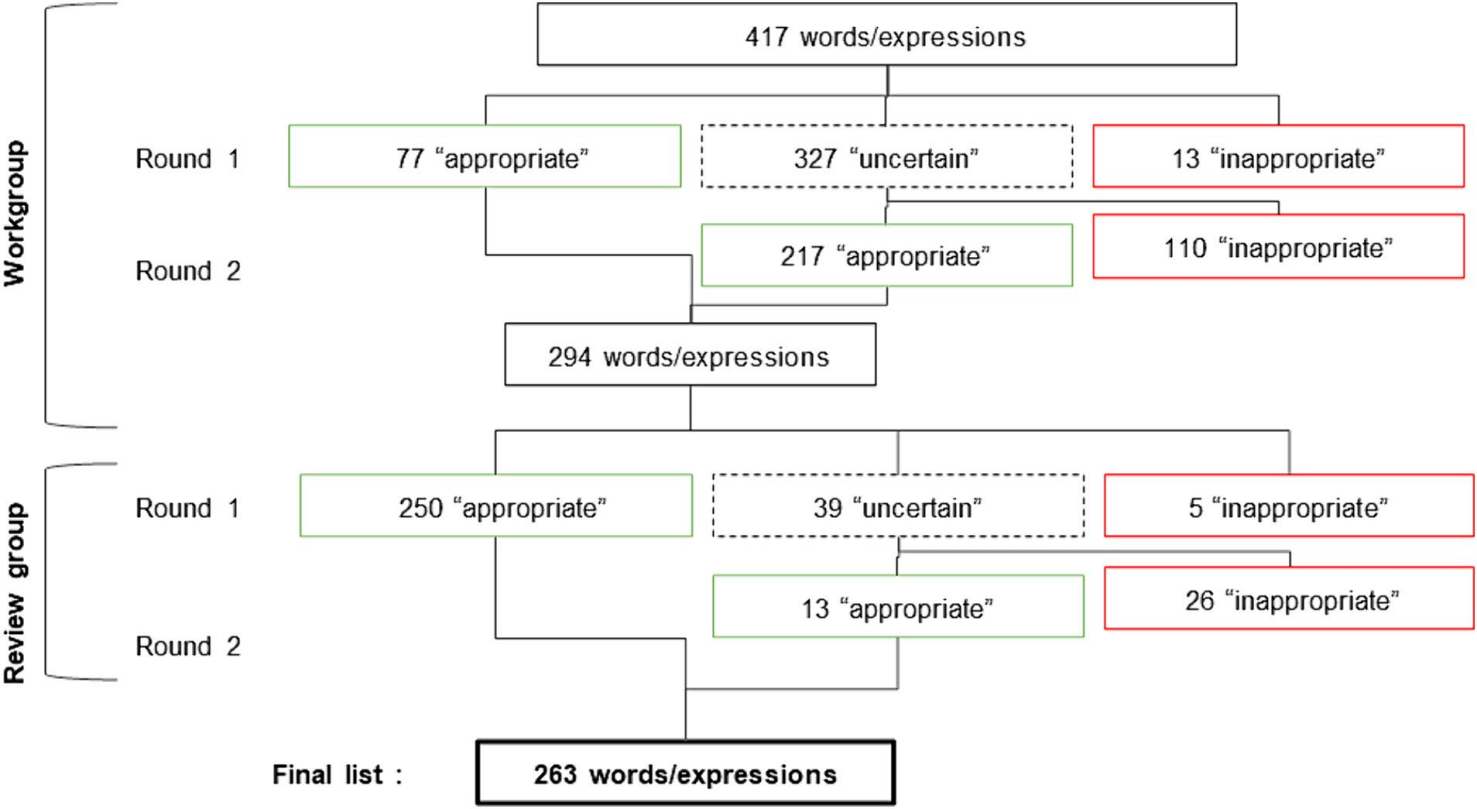
Flowchart of the selection of the words/expressions.

After pooling the list of words proposed by each of the six members of the working group, 417 words/expressions were ranked. After the first round of evaluation, 77 of them were rated as “appropriate,” 327 as “uncertain” and 13 as “inappropriate.” After discussion between the experts of the working group, the second round of evaluation permitted the elimination of 110 words/expressions rated as “inappropriate.” The remaining 294 “appropriate” words/expressions composed the list analyzed by the reviewers (Figure 4).

The review group rated each of these 294 words/expressions according to the formalized expert consensus (Figure 3). During the first round, they rated 250 words/expressions as “appropriate,” 39 as “uncertain” and deleted five words/expressions. During the second round, 13 of the 39 “uncertain” were discussed until agreement. Finally, a list of 263 words/expressions were given to the working group who defined them (Figure 4).

### 3.2 Results of the process leading to the list of definitions associated to words/expressions

**Figure 5:**
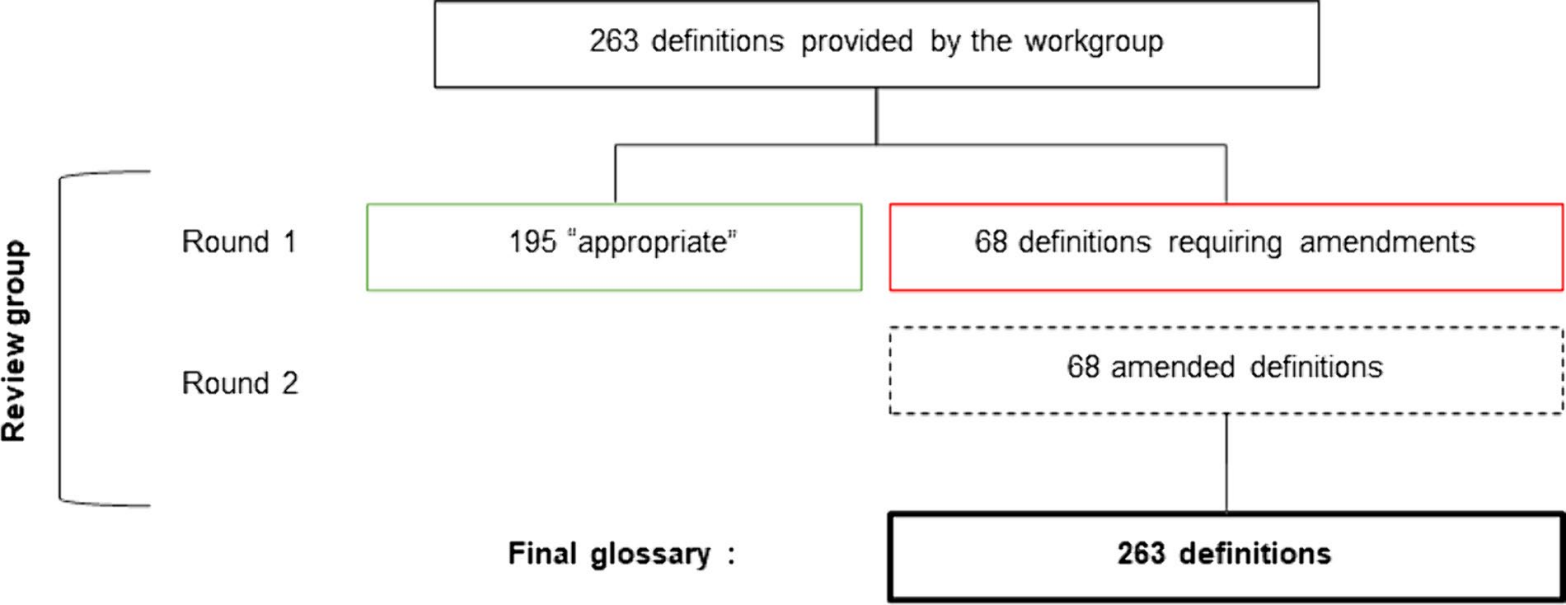
Flowchart of the validation of definitions by the review group.

The definitions of the 263 words/expressions were rated by the review group. The first round of evaluation established 195 definitions as being appropriated, and 68 definitions as requiring amendment. The 68 definitions were amended by the review group (Figure 5).

### 3.3 Example of definition

Table 2 presents an example of a definition which was translated from French to English.

**Table 2:** Example of the definition of “Health pathway” translated from French to English.

**Health pathway** Agreement in the workgroup: appropriate – strong agreement 9 (7–9) Agreement in the review group: appropriate – relative agreement 7 (5–9) **Etymology** Health: Latin sanitas, −atis, from sanus, healthy. Pathway: Latin percursus, with the influence of course. **Current dictionary definition** Pathway: all the stages through which something passes. Health: state of the body, good or bad. **Definition from an official source** “Today, a pathway is understood to include the comprehensive, structured and continuous management of patients, as close to home as possible. This calls for a major shift in our healthcare system to bring together prevention, care, medical-social and social follow-up. To put it plainly, we need to promote the emergence of “primary care” and support the “ambulatory shift” needed to improve the gradation of care…. health care pathways, which combine care with upstream health and social prevention, and downstream medico-social and social support, as well as homecare and return-to-work services.” Source: French ministry of health and solidarity. **Definition from an academic source** “Health pathways result from the coordinated delivery of health and social services to meet people’s prevention and care needs, within a framework of controlled expenditure. To achieve this, professionals need to organize themselves in such a way that the right services are delivered to the right patients, at the right time, by the right professionals. The organization of care paths must enable the “appropriate or relevant” implementation of healthcare interventions, guaranteeing effectiveness, safety and patient satisfaction, as well as efficiency, equity, accessibility and continuity of care.” Source: French graduate school of public health. **Definition from the field** “A health pathway is understood to mean comprehensive, structured and continuous care for patients, as close to home as possible. This entails bringing together prevention, care, medico-social and social follow-up, and moving away from a sector-by-sector approach. The work of health and social professionals is therefore coordinated, concerted and decompartmentalized.” Source: Regional health agency of the region grand Est. Associated terms: health, care pathway, pathway of life, public health.

## 4 Discussion

### 4.1 Main results

The outcome of this collective work is a glossary about the field of health pathways composed of 263 words/expressions defined by different sources. The creation of a glossary on healthcare pathways offers several advantages and addresses various needs in the healthcare field. Firstly, it allows for the definition of specific terms as the field of care pathways can be complex and involve the use of specialized terminology. A glossary provides definitions for technical and medical terms, thus facilitating understanding among different actors. Second, by establishing an official list of terms and their definitions, it contributes to the standardization of terminology used in the field of care pathways, promoting communication among different healthcare actors, which is essential for ensuring quality care and effective coordination. Third, it enhances transdisciplinary communication and understanding among various actors in healthcare pathways (physicians, nurses, social workers, therapists, etc.). Fourth, it serves as a reference for academic and clinical research, as well as for teaching in healthcare institutions and universities.

The glossary is aimed at several audiences: students from the health sector, amongst which PhD candidates; trainees and interns working with research laboratories; the medical, paramedical and scientific professionals collaborating on the management of particular diseases or patient population; the patients and their caregivers wishing to learn more or to improve their level of literacy concerning the health pathways.

In order to disseminate this glossary and make it accessible, the communication medium could be a book or an e-book from a publisher, a dedicated website, etc. However, whatever format you choose, the words will be classified in alphabetical order. A table of content will be placed upstream. For the paper format, the page number corresponding to each definition will be indicated. For the digital format, links will be created so that users can click on the word and access it directly. In addition, in this format, for each definition, if words are defined in the glossary, clicking on them will allow direct access to their definition.

The strengths of this study are the panel of the experts from the research team with several levels of expertise and seniority, who volunteered to participate in the elaboration of the glossary, and the replicable and formalized methodology of expert consensus.

### 4.2 Limitations

This study has several limitations. Firstly, although the methodology used to create this glossary is replicable and can be used in all countries, it is specific to the French healthcare system. Some words/expressions do not exist in other countries or the definitions are not universal and transferable to other countries. Second, the working group and the review group were set up on a voluntary basis, so the working group included only one practitioner specialized in neurology. However, other laboratory members were interviewed from time to time. Third, experts cannot be sure that all the words/expressions related to health pathways are embedded in the glossary (non-exhaustiveness) and that some words belong to common language without specific definition (examples: personal medical record or medical management). Fourth, as any work that produces up-to-date data, another limitation is to keep updated definitions, especially those relating to legal texts.

### 4.3 Comparison with prior work

Direct benefits on the research team were observed. After months of being close to one another, the 13 members have gone to know each other personally. This enabled them to pool their specific expertise, to debate about the scope and perimeter of the concepts, to finally find a consensus and speak the same language. On a human level, this project has been a unifying force for the members who learned from their differences. This work has given rise to other research projects currently underway.

Other initiatives have also used the Delphi formalized expert consensus to elaborate a glossary of health terms. For example, a Consensus Paper on Terminology for use in the treatment of conservative spinal deformities based on the Delphi method was used to reach a preliminary consensus before the meeting, where the terms that still needed further clarification were discussed (12). Also, an international consensus of experts was found using a Delphi study technique in 3 rounds to assess agreement and then resolve disagreement on Hidradenitis Suppurativa definitions among international experts (13). A face-to-face consensus meeting was held between Delphi survey rounds two and three in the elaboration of the semantics in the active surveillance for men with localized prostate cancer. Bruinsma et al. presented results of this research project (14) and North et al. the glossary of neurostimulation terminology (15).

## 5 Conclusion

This glossary supports transdisciplinary communication, reduce the extent of variations in practice and optimize decision-making. International debate on all aspects might be strengthened by an improved understanding of the concept of health pathway.

It is the first time, to our knowledge, a research team has elaborated a glossary of the health pathway, with the approved methodology of expert consensus. The glossary gained consensus across a panel of senior and professional experts and represents a transdisciplinary work. Future work is needed to address issues such as updating the definitions, and to develop international consensus about the words/expressions and definitions.

## Data availability statement

The raw data supporting the conclusions of this article will be made available by the authors, without undue reservation.

## Author contributions

LF: Investigation, Methodology, Writing – original draft. EV: Investigation, Writing – review & editing. H-MS: Investigation, Writing – review & editing. MG: Investigation, Writing – review & editing. CK: Investigation, Writing – review & editing. CC: Investigation, Writing – review & editing. AD-B: Investigation, Writing – review & editing. SR: Investigation, Writing – review & editing. CD: Conceptualization, Supervision, Writing – review & editing. GM: Conceptualization, Supervision, Investigation, Writing – review & editing. FC: Conceptualization, Investigation, Supervision, Writing – original draft.
